# 

**Published:** 1841-05

**Authors:** 


### THE WESTERN JOURNAL OF MEDICINE AND SURGERY:

EDITED BY
DANIEL DRAKE, M. D.
AND
LUNSFORD P. YANDELL, M. D.
PROFESSORS IN THE LOUISVILLE MEDICAL INSTITUTE.

NO. XVII.—MAY, 1841.
VOL. III.—NO. V.

LOUISVILLE, KY.
PUBLISHED MONTHLY, BY PRENTICE & WEISSINGER,
PROPRIETORS AND PRINTERS.
1841.

*This Number contains 4 sheets—Postage under 100 miles 6 cents, over 100 miles 10 cents.*

